# Investigating the Impact of Fear Related to COVID-19 Disease on Situational Humor *via* Social Networking Sites

**DOI:** 10.3389/fpsyg.2021.721304

**Published:** 2021-09-24

**Authors:** Ying Xue, Sajid Hassan, Sher Bahader, Shazia Habib

**Affiliations:** ^1^School of Management, Wuhan Polytechnic University, Wuhan, China; ^2^Clinical Psychology, International Islamic University Islamabad, Islamabad, Pakistan; ^3^Department of Applied Psychology, Government College University Faisalabad, Faisalabad, Pakistan

**Keywords:** social networking sites, situational humor, fear of COVID-19, university students, education

## Abstract

The current study investigates the impact of fear related to coronavirus 2019 (COVID-19) disease on situational humor, and also on social networking sites (SNS) usage as a mediator. Furthermore, this study investigates the impact of fear-related COVID-19 disease on situation humor *via* SNS usage, focusing on the gender perspective of university students. This study also examined the impact of fear related to COVID-19 disease on situational humor in students from various degree programs, such as BS and MS. For this cross-sectional study, data were collected from 24 different Pakistani universities using purposive sampling between December 2020 and May 2021. This study used social media platforms like WhatsApp, Facebook, LinkedIn, and Twitter (and also email) to collect data online. According to the findings of this study, fear related to COVID-19 disease was found to be significantly negatively related to situational humor and SNS usage, whereas SNS usage was positively related to situational humor. The findings also revealed that SNS usage is a key mediator in the relationship between fear related to COVID-19 disease and situational humor. In addition, male university students used more SNS and had a higher situational humor response than female university students, and female university students reported a higher fear related to COVID-19 disease. This study provided useful findings of the impact of fear related to COVID-19 disease on situational humor among students in various academic programs, such as bachelor's program and master's program.

## Introduction

The coronavirus 2019 (COVID-19) pandemic is a serious threat to the worldwide society, affecting every aspect of life and continuing to pose a challenge to global nations. Since COVID-19 became a pandemic, the focus of the world has properly been on protecting the public, preventing virus transmission, and treating victims of COVID-19 (Kumar et al., 2021). During this time, governments all over the world have enacted rules to minimize human contact, such as quarantine (Lin et al., 2020), physical separation, and social isolation. Following the first confirmed case on February 26, 2020, in Pakistan, the government has implemented several measures, namely, mandatory online learning in all schools, mandatory quarantine for people under the age of 18 and over the age of 65, 15-day quarantine in state accommodation units for people traveling from abroad, mandatory face mask use outside, and a mandatory vaccination program (Khattak et al., 2020). Although these efforts are necessary to stop the spread of COVID-19, they must be weighed against the possibility of detrimental mental health outcomes. Individuals who have been quarantined may develop boredom, hostility, and fear related to COVID-19 (Zheng et al., 2020). As a result of the symptoms of the viral infection mixed with the unfavorable effects of treatment, cognitive distress and anxiety of people may intensify (Khan et al., 2021).

The COVID-19 pandemic has evolved into the most serious public health issue of the world, posing a major threat to people everywhere. Apart from the immediate danger of the virus, the current scenario has caused a great deal of stress, and also enormous anxieties and concerns about the virus, which has had significant social and economic effects on the health and activities of people (Khan, 2021). COVID-19-related anxieties and social alienation could be a source of stress and disease fear (Ahorsu et al., 2020). Furthermore, fear related to COVID-19 causes people to remain on high alert to protect themselves and their loved ones, which can lead to social isolation, fear, and panic (Yip and Chau, 2020). Furthermore, digital and social media are among the sources that are propagating panic about the COVID-19 pandemic by disseminating sensational and false news and stories, which has raised stress and anxiety in people (Zheng et al., 2020). During the ongoing COVID-19 outbreak, on the other hand, the usage of social networking sites (SNS) platforms has provided a welcome escape from health disasters and global disasters (Cao et al., 2018). According to the U.S. Census Bureau, more than 42% of persons who used SNS in December 2020 showed signs of depression and anxiety, up 11% from the previous year (Olelewe, 2020).

The education sector is the most affected worldwide by the COVID-19 epidemic, which causes students to stay at home owing to school, college, and university closures. They mostly rely on online classes to continue their academic pursuits, and social media is their primary means of bridging the communication and interaction gaps that exist between face-to-face students at schools and universities (Petzold et al., 2020). The COVID-19 pandemic has had a variety of effects on students. For example, fear and anxiety are generated by staying at home and implementing protective measures such as social isolation, on the one hand (Durante et al., 2021). Students, on the other hand, were stressed out due to the uncertain nature of their studies and exams (Zheng et al., 2020). This situation left the students in a panic, and it strengthened their reliance on SNS and digital media for communication and instructional activities (Lin et al., 2020). In previous studies, in the COVID-19 situation, the usage of SNS is rarely investigated in the context of the mental health of students (Steinfield et al., 2008). Fear related to COVID-19 is linked to intolerance of instability (Cao et al., 2019), health suffering, the risk to loved ones, and expanded information channels (e.g., traditional media, SNS usage, and professional media) (Lin et al., 2020). People are also concerned about the effects of COVID-19 on the healthcare system, the economy, culture, job loss, and changes in daily routines, all of which may add to the problem (Durante et al., 2021).

**Figure 1:**
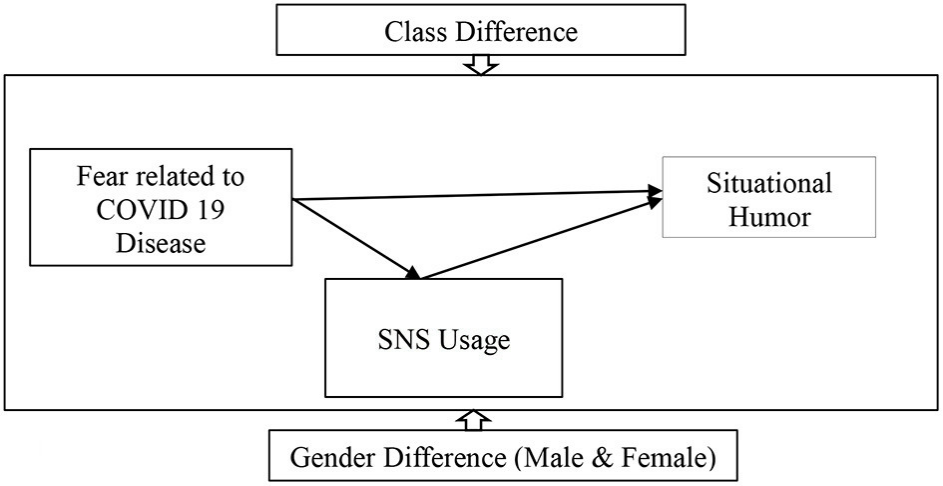
Research model.

The purpose of this study is to see how fear related to COVID-19 affects the situational humor of students and their usage of SNS. Furthermore, this study looks at the role of SNS usage as a mediator in the indirect association between fear of the COVID-19 pandemic and student situational humor. In particular, this research looks into the impact of gender (male vs. female) and educational disparities on the predicted link in our research model. We anticipate that this research will contribute to a better understanding of the link between the dependent variable (fear related to COVID-19) and the independent variable (situational humor). On the other hand, this research will help us gain a better grasp of how gender differences affect these relationships. Similarly, understanding the effects of education differences on our suggested research model will be helpful (see Figure 1).

## Hypothesis Development

According to a large-scale study, the general populace of Iran has a very high dread about COVID-19 (Ahorsu et al., 2020). Fear, according to previous experience, can have a negative impact on the health of an individual (Lin et al., 2020), and also negatively impact individual happiness. Governments require data on COVID-19 fear among the general public to establish effective COVID-19 transmission control policies that do not increase psychological distress (Wang, 2021). Many public institutions, including education, are currently focusing their efforts and resources on controlling COVID-19 infection, and have implemented national policies to reduce the spread of the virus (Liang et al., 2020), such as social distancing measures, stay-at-home policies, online classes, and the closure of public educational institutions (Rapanta et al., 2020). Although these educational policies and practices may effectively control the growth of COVID-19, their implementation will likely increase fear among children and parents, causing psychological and educational difficulties (Khattak et al., 2020). The human nature of SNS usage is violated by such protracted stays indoors and without face-to-face contact with family and friends (Xue et al., 2020), which has disrupted the daily-living routines of persons, and heightened fear of infection and psychological stress. Students are in a similar scenario when propagating the fear of the COVID-19 pandemic (Cao et al., 2019). Similarly, students may experience a negative impact on their sense of humor as a result of spreading the fear of the COVID-19 outbreak. Thus, the following hypothesis is proposed in this study.

**H1:** Fear related to the COVID-19 pandemic among students has a negative impact on student situational humor.

Individuals who are compelled to stay at home for an extended period have little choice but to change their focus from social to indoor activities and may wind up relying more on SNS and the Internet for updated information, amusement, and virtual gossip with friends (Zheng et al., 2020). One reason for the rising use of the Internet and SNS usage is that people want to learn more about COVID-19. Indeed, after the emergence of COVID-19, more than 80% of the 1,304 participants in a recent Chinese study reported remaining at home 20–24 h a day due to school and company closures (Wang et al., 2020). According to the same study, more than 90% of participants learned about COVID-19 on the Internet and were eager to learn more about it, namely, the COVID-19 transmission route, medication and vaccine availability and effectiveness, travel advice, overseas COVID-19 control experiences, the number of confirmed cases with locations, and COVID-19 prevention advice. Liu et al. (2021) found that COVID-19 information overload *via* SNS usage seemed to have a detrimental effect on the mental well-being of general SNS usage. According to mental health literature, COVID-19 affects college students on many levels, such as fear of contracting a disease or being able to infect others in their SNS usage, anxiety about transformations in course content delivery, and uncertain classroom parameters (Tasso et al., 2021). Nabity-Grover et al. (2020) argue that global health catastrophes, like the COVID-19 epidemic, change how and what people share on SNS. The fear related to COVID-19 may have an impact on those who work in the field of SNS and the Internet. Fear of a COVID-19 pandemic is likely to have a negative impact on SNS usage. The following hypothesis is proposed in this study.

**H2:** Fear related to the COVID-19 pandemic among students has a negative impact on the usage of SNS.

The tendency of experiences to make others laugh and amuse them is known as humor. Humor was found to engage and sustain the interest and attention of students when used by human educators (Zillmann et al., 1989). Unfortunately, disease information obtained from the SNS is not always reliable. Participants in a recent study analyzing general public awareness and attitudes of COVID-19 in the United States and the United Kingdom believed several misconceptions and falsehoods that had proliferated on SNS usage (Geldsetzer, 2020). Although many mental health professionals and public health experts have stated that psychological distress among different populations should be considered during the COVID-19 outbreak (Lin et al., 2020) to the best of the knowledge of the present authors, only three studies have collected empirical data on this topic (Ahorsu et al., 2020). The significance of SNS usage and the Internet in linking psychological processes and their impact on individual personality traits has been highlighted in several previous research (Khan and Khan, 2019; Wang et al., 2020). According to the WHO, students are currently fighting not only a global pandemic (COVID-19) but also an SNS epidemic, with some media outlets claiming that the coronavirus is the first true SNS antidepressant because it has infected the entire globe. People are panicking and fearful because of the rapid flow of information and misinformation (Hao and Basu, 2020). According to Sarner (2020), hearing a lot of information and news about COVID-19 has influenced the public and spread fear. According to Naeem (2021), fear related to COVID-19 spreads more quickly among SNS users than among those who use less SNS. People rely on SNS for COVID-19 information and facts because some countries use filters, which is why SNS provides fear related to COVID-19 but all the facts. After all, SNS users use the platform to express their feelings, emotions, and thoughts, which can be a valuable source of data for mental health research (Cellan-Jones, 2020). In this regard, the Internet and SNS usage could play a role in the relationship between COVID-19 fear and situational humor. To predict the mediating role of SNS usage, this study suggests the following hypothesis.

**H3:** The relationship between fear related to COVID-19 disease and situational humor is mediated by SNS usage.

Fear is one of the most common psychological repercussions of COVID-19, as seen above. In extraordinary circumstances, such as illness outbreaks and epidemics, people appear to be terrified (Pakpour and Griffiths, 2020). COVID-19 fears are likely to be fueled by the first appearance and the uncertainty of disease surrounding its future (Asmundson and Taylor, 2020). Several previous studies have looked at how fear affects humor and happiness in students and employees, but few have looked at how fear of the COVID-19 pandemic affects situational humor among university students by gender. To assess the effect of gender differences on the relationship proposed in our research model, this study offered the following hypothesis.

**H4:** Fear related to COVID-19 disease has a higher impact on SNS usage and situational humor of female students rather than male students.

Furthermore, the previous study found that over usage of SNS the link between scarcity messages and perceived excitement, whereas the desire to buy moderates the relationship between perceived excitement and behavior impulsively (Islam et al., 2021). Moreover, the global COVID-19 catastrophe has wreaked havoc on the global economy and medical care, leaving many thousands of people (including students) in a state of fear, panic, and uncertainty (Islam et al., 2021). In the previous research, it was found that brand communication had a significant impact on the relationship between consumer engagement and brand attachment on SNS usage (Arya et al., 2018). This study proposed the following hypothesis to evaluate the impact of the above variables in the setting of educational groups in the current research.

**H5:** Fear related to COVID-19 disease has a higher impact on SNS usage and situational humor of students of BS program rather than students of MS program.

## Method

### Data Collection Procedures

A pilot study with a small sample size was conducted before the full-scale study. The purpose of the pilot study was to ensure that the variables and construct items proposed in our research model were both reliable and valid. A total of 50 students were contacted *via* social media platforms at their convenience, but only 30 of them responded completely. The majority of respondents (64%) were male, and the majority of students (79%) were undergraduates.

This full-scale study was quantitative and cross-sectional. The survey of this study took place between December 2020 and May 2021, with a sample of students from Pakistani universities. A Google Forms-based online survey questionnaire was created to collect responses. The COVID-19 situation in Pakistan continued to deteriorate during this period, with 139–160 cases recorded each day, gradually escalating to several hundred cases per day (Abid et al., 2020). This study approached the students of 24 universities (BS students, *f* = 417, % = 34.8; MSc students, *f* = 553, % = 46.1, MS students, *f* = 136, % = 11.3; and PhD students, *f* = 94, % = 7.8). Prospective participants were given an explanation sheet that includes details regarding the study (such as the title, the purpose and significance of the study, privacy information, and the email addresses and phone numbers of researchers). The community circulated the informational document to other students in person and by email, text message, and SNS usage. Students who agreed to participate were approached by email or text message by a member of the study team. The researchers answered any queries these potential participants had concerning the study. Each student who accepted to participate received a survey that directed him to the consent form, and their agreement was confirmed with an electronic signature (i.e., the ticking of a box on the form). After getting consent, participants were texted or emailed a link to the Google Forms questionnaire. The online questionnaires were distributed to 1,500 university students, and 1,200 (90%) responded. The following criteria were used to decide who was eligible to participate: (1) enrolling at a university and (2) a Pakistani citizen. Students who were enrolled in colleges or institutions were not allowed to participate. Purposive sampling was used in this study because it focuses on certain characteristics of a population that are of interest. In this context, university students whose academic activities were hampered were sampled, and they were using SNS to get updates and the latest news about the COVID-19 pandemic situation, and educational instruction from universities and educational authorities. The online questionnaire was created using Google Forms. Google Forms is an online application that allows you to collect feedback from users quickly and efficiently. The survey results were automatically gathered in an Excel spreadsheet and then imported into SPSS for analysis.

### Measurement Scale

This study used a measurement scale that had been used in previous studies. Three authentic and reliable instruments were used in this study to collect participant responses: the Fear Related to COVID-19 Scale, the Situational Humor Response Questionnaire, and the Social Networking Sites Usage and Needs Scale. These three scales were used in both the pilot and the main study. These scales have been used for a pilot study.

#### Fear Related to COVID-19 Scale

The COVID-19 Fear Scale was a seven-item questionnaire designed by Ahorsu et al. (2020) to measure COVID-19 fear in the general public. On a five-point scale, 1 = “strongly disagree,” 2 = “disagree,” 3 = “neutral,” 4 = “agree,” and 5 = “strongly agree.” Higher scores indicate a higher level of fear related to COVID-19, whereas low scores indicate a low rate of COVID-19 fear. The scale has sufficient concurrent validity; nonetheless, the Fear of COVID-19 Scale has Cronbach's alpha value of 0.82 and test-retest reliability of 0.72, according to the authors. For this study, Cronbach's alpha was 0.82.

#### Situational Humor

This study used 21 items situational humor response questionnaire adopted by Martin (1996) to measure situation humor. This scale has 21-item scale use to test the sense of humor of people in specific situations. This scale was rated on a five-point Likert scale ranging from 1 (strongly disagree) to 5 (strongly agree) and Cronbach's alpha is 0.93.

#### SNS Usage

This study used Social Network Sites Usage and Needs Scale adopted by Ali et al. (2020). This scale contains 29 items and minor changes were made in scale statements to relate to the particular context of COVID-19. All statements were scored on a five-point Likert scale ranging from 1 (strongly disagree) to 5 (strongly agree) and Cronbach's alpha is 0.91.

## Data Analysis

The data were entered and analyzed using SPSS software (Version 24). The data were checked for missing information and outliers first. We did not have any missing data since each question on the e-survey had a star next to it, and participants could not go on without answering the preceding question. Outliers were visually inspected for scattered plot diagrams, but no outliers were found. Frequencies (*n*), percentages (%), means (*M*), SDs, and median were utilized as descriptive statistics. Inferential statistics approaches such as independent samples *t*-tests and variations among demographic subgroups were used to categorize differences in demographic data. The association between the outcome variables (SNS usage, situational humor, and fear related to COVID-19) was further investigated by using Pearson's moment correlation, multiple linear regression, mediation analysis, *t*-test, and the one-way ANOVA. The statistical significance level was set to *p* < 0.001.

### Reliability Test

Table 1 showed the reliability analyses of scales, with the SNS usage, possessed reliability of 0.79, indicating fascinating reliability. The situational humor had a reliability of 0.80, which was outstanding, and the Fear Related to COVID-19 Scale had a reliability of 0.88, which was exceptional in a pilot study.

**Table 1:** Cronbach's alpha reliability analysis of scales.

**Variables**	**K**	**A**
**Pilot study**
SNS usage	29	0.79
Situational humor	21	0.80
Fear related to COVID-19	7	0.88
**Full-scale study**
SNS usage	29	0.83
Situation humor	21	0.85
Fear related to COVID-19	7	0.82

*(N = 1,200); SNS, social networking sites*.

In this study, Table 1 showed the reliability analyses of scales in which the reliability of SNS usage was 0.83, which shows good reliability. Reliability of situational humor 0.85, which was also excellent reliability, and the reliability of Fear Related to COVID-19 Scale was 0.82 which was good reliability.

### Descriptive Statistics and Correlation

Correlations were computed to investigate the relationships between SNS usage, situational humor, and fear related to COVID-19 (see Table 2).

**Table 2:** Descriptive statistics and correlations for study.

**Variables**	**1**	**2**	**3**
**Pilot study**
*F-COVID-19*	–		
*Situational humor*	−0.60^**^	–	
*SNS usage*	−0.47^**^	0.57^**^	–
**Full-scale study**
*F-COVID-19*	–		
*Situational humor*	−0.56^**^	–	
*SNS usage*	−0.40^**^	0.37^**^	–

** *p <0.001; SNS, social networking sites*.

Table 2 revealed that pilot study indicates that fear related to COVID-19 was significant negatively associated with situational humor (*r* = −0.60, *p* < 0.001) and SNS usage (*r* = −0.47, *p* < 0.001). While situational humor was positively associated with SNS usage (*r* = 0.57, *p* < 0.001).

For full-scale study, fear related to COVID-19 was significantly negatively associated with situational humor (*r* = −0.56, *p* < 0.001) and SNS usage (*r* = −0.40, *p* < 0.001). SNS usage was positively associated with situational humor (*r* = 0.37, *p* < 0.001).

### Regression Analysis

Table 3 shows the impact of SNS usage and situational humor on fear related to COVID-19 in university students. The R2 value of 0.35 revealed that the predictors explained 35% variance in the outcome variable with *F*_(2,1, 197)_ = 321.84, *p* < 0.001. The findings revealed that SNS usage significantly predicted fear related to COVID-19 (β = −0.015, *p* < 0.001), whereas situational humor also significantly predicted fear related to COVID-19 (β = −0.014, *p* < 0.001).

**Table 3:** Regression coefficients of SNS usage and humor on fear related to COVID-19.

**Variables**	**B**	***SE***	***T***	***p***	**95%CI**
*Constant*	48.89	0.67	73.86	0	[47.69, 50.29]
*SNS usage*	−0.12	0.01	−8.47	0	[−0.16, −0.09]
*Situational humor*	−0.26	0.01	−19.0	0	[−0.27, −24]

*SNS, social networking sites*.

Table 4 shows the impact of SNS usage and situational humor on fear related to COVID-19 in university students.

**Table 4:** Regression analysis for mediation of SNS usage between situational humor and fear related to COVID-19 disease.

**Variable**	***B***	**95%CI**	***SE***	**β**	***R* ^**2**^**	**Δ*R*^**2**^**
Step 1					0.31	0.31^***^
Constant	45.67^***^	[44.59, 46.74]	0.56			
Situational humor	−0.30^***^	[−0.33, −0.28]	0.01	−0.56^***^		
Step 2					0.35	0.04^***^
Constant	47.99^***^	[47.69, 50.30]	0.66			
Situational humor	−0.26^***^*	[−0.29, −0.24]	0.014	−0.48^***^		
SNS usage	−0.12^***^	[−0.15, −0.95]	0.016	−0.21^***^		

*** *p <0.001; SNS, social networking sites*.

In step 1, R2 value of 0.31 revealed that situational humor explained 31% variance in the fear related to COVID-19 disease *F*_(1,1, 198)_ = 593.98, *p* < 0.001. The findings revealed that situational humor significantly negatively predicted fear related to COVID-19 disease (β = −0.56, *p* < 0.001). In step 2, the R2 value of 0.35 revealed that situational humor and SNS usage explained 35% variance in the fear related to COVID-19 with *F*_(1,1, 198)_ = 321.84, *p* < 0.001. The findings also verified that SNS usage (β = −0.48, *p* < 0.001) significantly negatively predicted fear related to COVID-19 disease (β = −0.21, *p* < 0.001). The ΔR2 value of 0.04 revealed 4% change in the variance of model 1 and model 2 with Δ*F*_(1,1, 198)_ = 71.80, *p* < 0.001. The regression weight for situation humor subsequently reduced from model 1 to model 2 (−0.56 to −0.48) but remained significant which confirmed the partial mediation. More specifically, situational humor has direct and indirect effects on SNS usage.

### Gender Perspective Mean Analysis

Table 5 revealed significant mean differences on SNS usage with *t*_(1,198)_ = 3.56, *p* < 0.001. Findings showed that male students exhibited higher score on SNS usage (*M* = 42.84, *SD* = 2.61) as compared to female students (*M* = 41.67, *SD* = 2.67). The value of Cohen's *d* was 0.20 (=0.20) which indicated a small effect. The findings revealed significant mean differences in situational humor with *t*_(1,198)_ = 3.87, *p* < 0.001. In addition, male students exhibited higher score on situation humor response (*M* = 41.78, *SD* = 2.66) as compared to female students (*M* = 41.56, *SD* = 2.93). The value of Cohen's *d* was 0.22 (<0.40) which indicated medium effect.

**Table 5:** Mean comparison of male students and female students on SNS usage, humor, and fear related to COVID-19 disease.

	**Male**		**Female**				
**Variables**	***M***	***SD***	***M***	***SD***	***t*_**(3, 12)**_**	***P***	**Cohen's *d***
SNS usage	41.84	2.61	41.29	2.67	3.56^***^	0	0.204
SH	41.78	2.66	41.56	2.92	3.87^***^	0	0.229
FCV-19	32.78	1.47	33.09	1.61	−3.84**	0.001	0.201

*** * indicate the higher significance value of variables*.

Table 6 showed the mean, standard deviation, and *F* values for SNS usage, situational humor, and fear of COVID-19 disease across education groups. Results indicated significant mean differences across education groups on SNS usage *F*_(3,1, 197)_ = 2.67, *p* < 0.001. Findings revealed that MSc students exhibited higher scores on SNS usage as compared to the others three education groups (BS students, MS students, and PhD students). The value of Cohen's *d* was 0.44 (<0.50) which indicated a small effect size. *Post-hoc* comparisons indicated significant between-group mean differences of each group with the other three groups.

**Table 6:** Mean, standard deviation, and one-way ANOVA in SNS usage, situational humor, and fear related to COVID-19 across education groups.

	**BS student**		**MSc student**		**MS student**		**PhD student**				
**Variables**	***M***	***SD***	***M***	***SD***	***M***	***SD***	***M***	***SD***	***F*_**(3, 12)**_**	**η*^**2**^***	***Post hoc***
SNSs	41.57	2.64	41.70	2.57	41.10	2.92	41.02	2.54	2.67^**^	0.44	2>1>3>4
SH	41.61	2.67	41.54	2.81	40.94	3.07	41.88	2.71	2.63^**^	0.46	4>1>2>3
FCV-19	32.98	1.51	32.87	1.54	33.12	1.73	32.54	1.25	2.10^**^	0.43	3>1>4>2

** * indicate the significance value of variables*.

Results indicated significant mean differences across education groups on situational humor *F*_(3,1, 197)_ = 2.63, *p* < 0.001. Findings revealed that PhD students exhibited higher scores on SNS usage as compared to the others three education groups (BS students, MSc students, and MS students). The value of Cohen's *d* was 0.46 (<0.50), which indicated a small effect. *Post-hoc* comparisons indicated significant between-group mean differences of each group with the other three groups.

Moreover, results indicated significant mean differences across education groups on fear of COVID-19 disease *F*_(3,1, 197)_ = 2.10, *p* < 0.001. Findings revealed that MS students exhibited a higher score on fear of COVID-19 disease as compared to the others three education groups (BS students, MSc students, and PhD students). The value of Cohen's *d* was 0.43 (<0.50), which indicated a small effect. *Post-hoc* comparisons indicated significant between-group mean differences of each group with the other three groups.

## Discussion

The study aimed to gain a better understanding of the impact of SNS usage in mediating the fear of a COVID-19 pandemic and situation humor. Although other researchers have looked into the influence of COVID-19 on individuals and employees (Cao et al., 2020; Zheng et al., 2020; Kumar et al., 2021), this study differs in that it studied the influence of COVID-19 on university students in a developing country (i.e., Pakistan) by looking at gender and class perspectives. Four hypotheses were examined in the study, namely, direct and indirect correlations, and also the hypothesized mediation model. COVID-19-related fear appears to be strongly linked to situational humor and SNS usage, whereas situational humor appears to be significantly linked to SNS usage. The results showed that fear related to COVID-19 significantly predicted humor and SNS usage, confirming our hypotheses based on the association between fear related to COVID-19 and situational humor, and also the association between fear related to COVID-19 and SNS usage. Overall, the findings of this study supported all of the proposed hypotheses, and they were consistent with the previous studies (Khan and Khan, 2021; Liu et al., 2021) in the related field.

### Theoretical Contribution

This study also adds to the literature on fear, particularly fear triggered by crises (such as COVID-19). The outcomes of this study support the previous research findings that show a detrimental relationship between personal fear, SNS usage, and humor (Lee et al., 2020). The findings of this study revealed a strong positive link between SNS usage and situational humor during COVID-19 crises, which is similar to a prior study that established a positive link between social media and individual humor in a normal setting (Yip and Chau, 2020). A mediating impact of SNS usage was hypothesized in the H3 indirect association between fear related to COVID-19 and situational humor, and the results supported a partial mediation. This differs from the previous research, which indicated that SNS usage fully mediated the relationship between individual anxiety and happiness. This distinctive finding could be the result of unfortunate conditions carried on by the COVID-19 pandemic. The impact predicted in H4 revealed significant mean gender differences in SNS usage, situational humor, and COVID-19 fear, according to the findings of this study.

As a result, this hypothesis has been proven correct: male students outperform female students in terms of SNS usage and situational humor. According to the findings of this study, female students had a higher fear of the COVID-19 disease than male students. In our society, men were more likely than women to use SNS, namely, blogs, media-sharing sites, social questioning, and humor user reviews. This finding could be explained by the fact that male students are less afraid of COVID-19 than female students, possibly because female students are unaware of daily ablates of COVID-19, resulting in a higher fear than male students. According to the past study, women were also more anxious, had more anxiety symptoms, and were more enamored of COVID-19 than men (Hou et al., 2020).

The impact of educational groups was predicted in the H5, and the results demonstrated significant between-group mean changes in the use of SNS, situational humor, and fear of COVID-19 of each group. Based on their educational level, we divided students into four groups: BS students, MSc students, MS students, and PhD students. The findings found that MSc students scored higher on SNS usage than the other three education groupings. When compared to the other three education groups, PhD students scored higher on situational humor (BS students, MSc students, and MS students). Furthermore, when compared to the other three education groups, MS students scored higher on fear of the COVID-19 disease. Overall, our hypotheses were partially supported by the literature.

### Practical Contribution

This study has several practical implications for educators, policymakers, and practitioners. First, this study emphasized the role of pandemic-related fear. This type of fear can have a negative impact on the sense of humor of students and their proclivity to the usage of SNS platforms to connect with like-minded individuals. It is suggested that university administration takes the appropriate steps to strengthen preventive measures among students rather than creating an environment of dread, which can influence not only their psychological health but also their academic performance. Second, the impact of SNS usage on students cannot be underestimated. Fake news and inappropriate usage of SNS, particularly SNS, can damage the sense of humor of an individual during the COVID-19 crisis. A sense of humor is thought to be beneficial in reducing stress. Students, teachers, and university management all take steps to ensure an effective online learning environment in this scenario. Furthermore, the university provides employees with useful online networking platforms where they may share positive news and gain confidence in the horrible condition created by COVID-19 disease. Third, online mentoring classes and counseling sessions for students, particularly undergraduate students, can be beneficial to them in terms of strengthening their situational humor and promoting their confidence and academic performance. Finally, understanding the fear associated with COVID-19 disease and its impact on SNS usage and humor is critical for university administration. Our findings show that female students are more effusive in this situation than male students. Female students, as a result, require more attention and positive measures in both physical and online classes to assure their participation.

### Limitation and Future Research Direction

There are some limitations to consider while analyzing the results. The study started with a nonclinical sample. Because clinical volunteers are more likely to experience persistent fear and despair during a pandemic, they should be used in the study. Second, due to the cross-sectional nature of the study, establishing a causal relationship from the data is difficult. Longitudinal and experimental studies are required to learn more about the causal relationships between the variables. In this study, data of these constructs (such as SNS usage, situational humor, and COVID-19) were collected all at once, hence, the chances of common method bias or social desirability may be ruled out. Third, data were gathered through the use of online and SNS applications, and also through email; however, poor Internet connections and a lack of detailed information may limit proper respondent responses; therefore, future studies can collect data physically. Finally, students who are exposed to potential macroaggressions such as ageism, ableism, classism, and racism may experience a conflation of preexisting stresses in addition to COVID-19. As a result, in future research, variables that may be useful in dealing with COVID-19 fear people in the COVID-19 process may be investigated in these specific groups rather than the general population. Finally, due to the nature of deliberate sampling, gender distribution equality was not reached. Future studies should look at gender distribution in the context of a nationally representative poll to tackle this challenge. Furthermore, future studies could solve this issue by utilizing meta-analysis.

## Conclusion

The current study indicated that COVID-19 fear was negatively connected with SNS usage and situational humor responses, but SNS usage was positively connected with situational humor. Fear related to COVID-19 of students was strongly influenced by their SNS usage and also how they reacted to humorous circumstances. Furthermore, the SNS usage was found to be a partial mediator between fear related to COVID-19 disease and situational humor in the mediation model. Furthermore, when compared to female university students, male students use much more SNS usage and respond to situations with situational humor, whereas fear of the COVID-19 disease of female students is significantly higher. It also found that individuals from different educational backgrounds had significant mean differences in study variables.

## Data Availability Statement

The data will be made available upon scholarly request via the first and second authors.

## Ethics Statement

This study and data collection procedures were approved by the Ethics Committee of International Islamic University (https://iiu.edu.pk/default.htm) and School of Management, Wuhan Polytechnic University (https://www.whpu.edu.cn/en/). The patients/participants provided their written informed consent to participate in this study.

## Author Contributions

YX and SHas developed research model and wrote the initial draft of this article including introduction, discussion, conceive of the project and oversees. SHas, YX, and SHab contributed data analyses. SHab and SB contributed in both revisions stages of the manuscript. All authors contributed to the article and approved the submitted version.

## Conflict of Interest

The authors declare that the research was conducted in the absence of any commercial or financial relationships that could be construed as a potential conflict of interest.

## Publisher's Note

All claims expressed in this article are solely those of the authors and do not necessarily represent those of their affiliated organizations, or those of the publisher, the editors and the reviewers. Any product that may be evaluated in this article, or claim that may be made by its manufacturer, is not guaranteed or endorsed by the publisher.
